# Autofusion With Magnetically Controlled Growing Rods: A Case Report

**DOI:** 10.7759/cureus.36638

**Published:** 2023-03-24

**Authors:** Michael J Yang, Alexander Rompala, Solomon Praveen Samuel, Amer Samdani, Joshua Pahys, Steven Hwang

**Affiliations:** 1 Orthopedic Surgery, Shriners Hospitals for Children, Philadelphia, USA; 2 Orthopedics, Tufts Medical Center, Boston, USA

**Keywords:** palsy, cerebral, rods, growing, controlled, magnetically, autofusion, surgery, spine, pediatric

## Abstract

Magnetically controlled growing rods (MCGRs) are an effective alternative to traditional growing rods (TGRs) in the treatment of early-onset scoliosis (EOS), with comparable deformity correction despite fewer planned reoperations. This case report presents a unique case of autofusion in a patient with tetraplegic cerebral palsy, thoracic myelomeningocele, and EOS who was treated with dual MCGR instrumentation and underwent serial lengthening procedures for four years. We detail the operative and radiographic findings in a novel case of autofusion encountered after MCGR placement to treat EOS. An eight-year-old female with tetraplegic cerebral palsy causing a 94° right thoracic neuromuscular scoliosis was treated with dual MCGRs; she then underwent serial lengthenings every four months. At 12 years of age, during MCGR explantation and posterior spinal fusion, dense heterotopic autofusion was encountered around the MCGR instrumentation, limiting further deformity correction. The benefits of MCGRs make them an appealing alternative to TGRs for the treatment of EOS. Although the theoretical risk of autofusion in MCGRs is low, recent case reports propose autofusion as a possible reason for MCGRs' failure to lengthen.

## Introduction

Early-onset scoliosis (EOS) is a complex spinal deformity that affects children under the age of ten [1]. Traditionally, growing rods have been used to treat EOS [1-4]. However, traditional growing rods (TGRs) have several limitations, including the need for repetitive surgical procedures for rod lengthening along with the associated repetitive anesthetic risks and the potential for complications such as infection, implant failure, and loss of correction [1-10]. In addition, growing rods have been associated with a high incidence of autofusion, which can limit further spinal correction and lead to long-term functional limitations [3,11].

To address the limitations of TGRs, magnetically controlled growing rods (MCGRs) have been developed. MCGRs allow for non-invasive rod lengthening, reducing the need for repetitive surgical procedures [12,13]. Moreover, MCGRs have been shown to reduce the incidence of complications associated with TGRs, including implant failure and infection [14]. However, there is limited information on the incidence of autofusion in patients with MCGRs, particularly in those who undergo repeated lengthening procedures [12-14].

In this case report, we present a case of autofusion in a patient with tetraplegic cerebral palsy and thoracic myelomeningocele who underwent placement of dual MCGR instrumentation. We also discussed the implications of autofusion in the management of spinal deformities and the role of MCGRs in reducing the incidence of complications associated with TGRs.

## Case presentation

**Figure 1:**
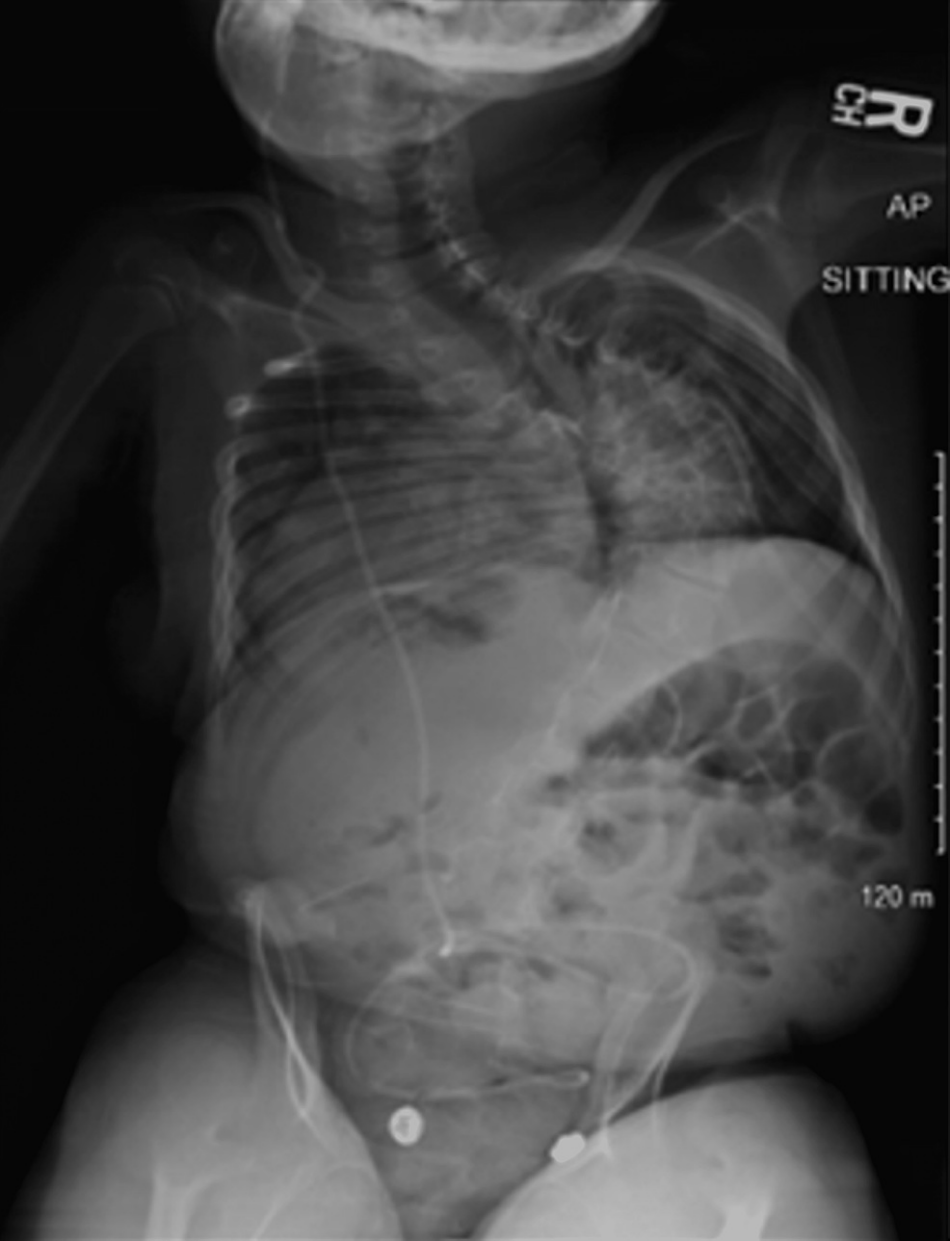
Posterior-anterior plain radiograph performed before index surgery for MAGEC rod placements at eight years of age.

**Figure 2:**
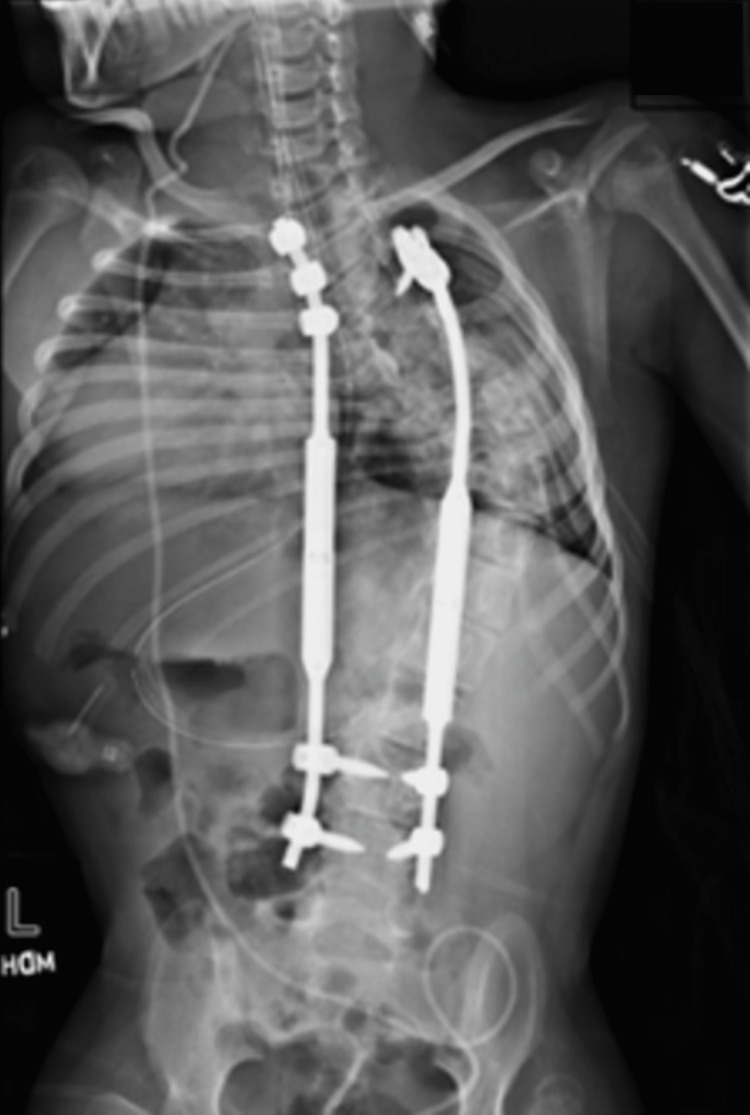
Posterior-anterior plain radiograph performed after index surgery for MAGEC rod placements at eight years of age.

An eight-year-old girl with tetraplegic cerebral palsy and thoracic myelomeningocele, who was primarily wheelchair-bound with significant spasticity and motor deficits that resulted in right thoracic neuromuscular scoliosis of approximately 94°, underwent dual magnetically controlled growing rods (MCGR, Nuvasive®, San Diego, CA, USA) instrumentation placement from T3 to L5 (Figures 1, 2). The procedure and her post-operative recovery were uneventful and imaging in the immediate post-operative period demonstrated a 20° correction to a 74° curve. Her MCGRs were lengthened every four months for a total of 11 lengthenings and monitored clinically and radiographically until she was 12 years old and had reached a total correction of 21° to a 73° curve, at which point a conversion to a posterior spinal fusion was planned since the actuator was no longer lengthening.

**Figure 3:**
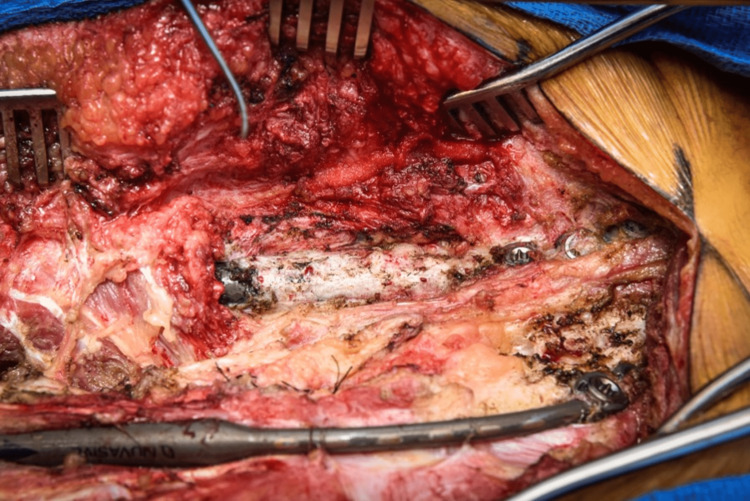
Intra-operative clinical photo depicting heterotopic bone formation around MAGEC rod placements at 12 years of age.

During the surgical procedure, when the implanted MCGRs were exposed, it was found that there was heterotopic bone formation surrounding both the rostral and caudal fixation points (Figure 3).

**Figure 4:**
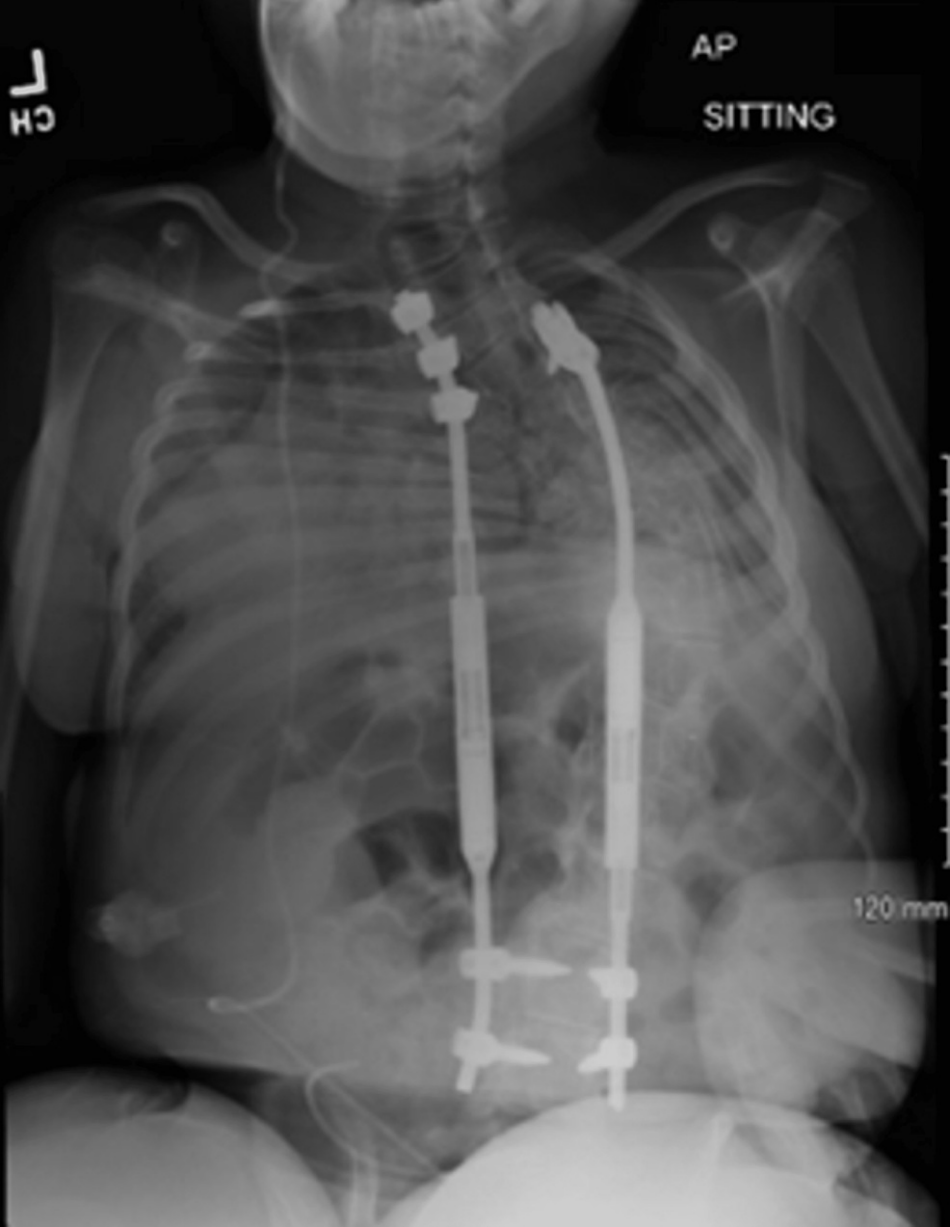
AP plain film performed before conversion surgery from MAGEC rods to fixed cobalt chrome rods.

**Figure 5:**
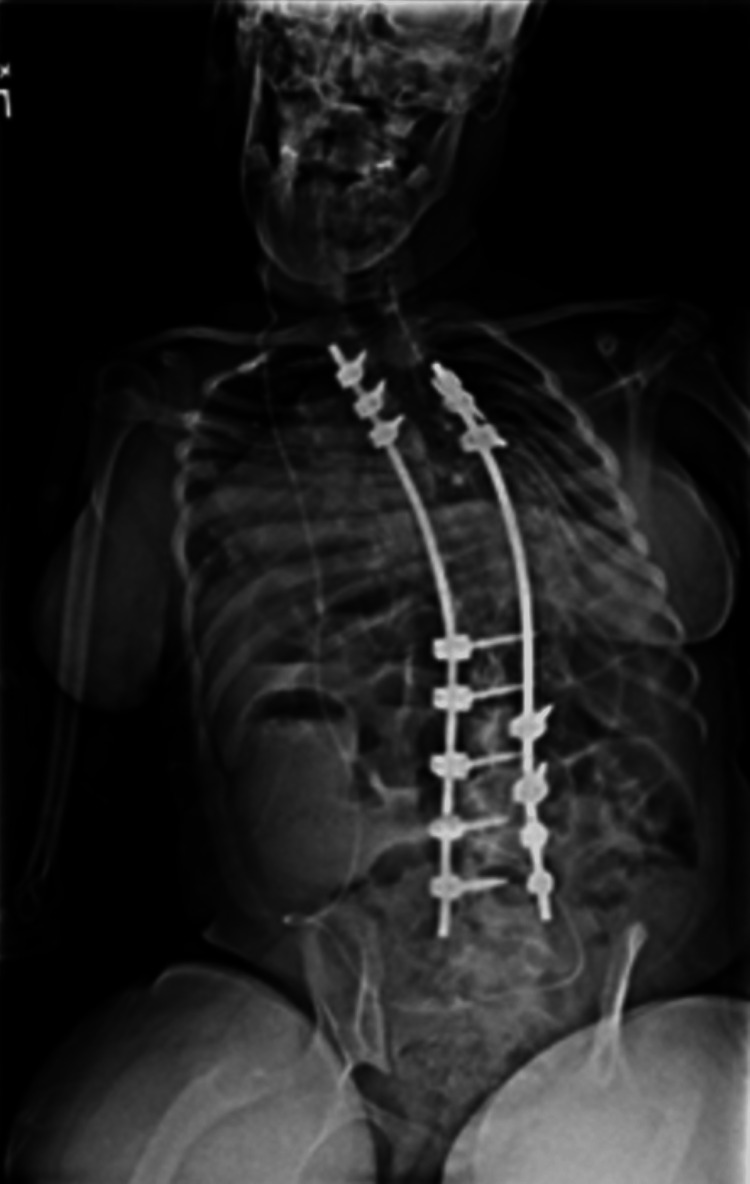
AP plain film performed after conversion surgery from MAGEC rods to fixed cobalt chrome rods.

The heterotopic bone was removed along with the MCGRs. It was discovered that there was underlying solid bony autofusion, which made it unnecessary to attempt any releasing osteotomies for further deformity correction. Instead, additional screws were placed, their location was confirmed with intra-operative navigation, and cobalt chrome rods were molded and secured for definitive posterior spinal fusion demonstrating a total correction of 40° to a final curve of 54° (Figures 4, 5).

Around three weeks after the surgery, there was minimal serosanguinous drainage from the posterior spinal incision with no associated fever or elevated lab values. After a week course of Keflex, the patient reported worsening wound dehiscence and drainage and was admitted for irrigation and debridement. She was subsequently treated with oral levofloxacin after cultures were positive for levofloxacin-sensitive Pseudomonas aeruginosa and Citrobacter freundii. Following a two-week course of levofloxacin, the incision then healed well without any further issues.

## Discussion

The treatment of progressive EOS is challenging given the balance that must be achieved between maximizing truncal growth and minimizing rotational deformity [1]. EOS management algorithms are highly variable, from bracing to early spinal fusion [1,4]. Each treatment has its drawbacks. Bracing may fail to prevent scoliotic progression, and compliance may limit its benefit [4]. In contrast, premature spinal fusion may cause reduced truncal height, predispose the immature spine to the crankshaft, and decrease thoracic volume leading to thoracic insufficiency syndrome [4-6].

In this context, TGRs were developed with the goal to reduce the progression of scoliotic deformity while simultaneously allowing truncal growth and thoracic cage expansion [1]. TGRs have been shown to effectively treat EOS, with an average total correction between 61% and 64% [1,3,4,7,8]. However, they necessitate repeat surgeries for serial lengthenings, which have been associated with an increased risk of infection, hardware failure, neurodevelopmental concerns regarding the need for multiple anesthetic sessions, and the socioeconomic burden of frequent surgery [9,10].

In addition, autofusion occurs with TGRs, which are thought to be secondary to immobilization, trauma to local paraspinal musculature, and the tendency of immature bone to heal stressed areas in the immature spine [3,11]. In their series of nine patients treated with TGRs, Cahill et al. found an 89% rate of autofusion [3]. Similar to premature posterior spinal fusion, autofusion associated with TGRs has the potential to limit scoliotic correction and reduce truncal growth [3].

MCGRs were developed in response to these drawbacks of TGRs. MCGRs can be serially lengthened in the outpatient clinic setting without the need for repeat anesthetic sessions and serial operations, thus reducing repetitive tissue trauma and theoretically the resulting high rate of autofusion. Satisfactory deformity correction with MCGRs, in addition to maintained flexibility for further correction at the time of definitive spinal fusion, is well documented [12,13]. MCGRs also have not been found to be limited by the “law of diminishing returns” associated with TGRs, as decreased lengthening with more distraction episodes has not been demonstrated [14].

MCGR treatment plans are not without risk. They have a higher hazard of unplanned revisions compared to posterior spinal fusions in patients with EOS [15]. A systematic review on the use of MCGRs for treatment of EOS found that, although there was a coronal improvement of 29.9° with maintained growth progression, this is paired with a mean complication rate of 44.5%, primarily from anchor pull-out, implant failure, or rod breakage [16]. MCGRs have a comparable complication rate and health-related quality of life outcome compared to TGRs [17].

**Figure 6:**
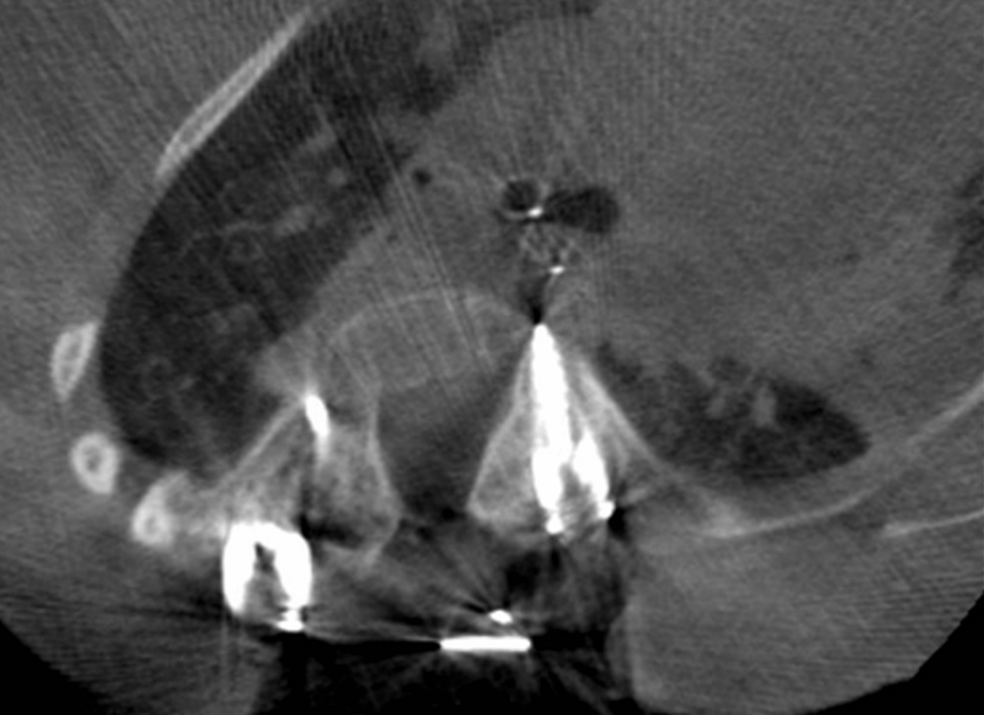
Superior axial intra-operative CT image illustrating heterotopic bony fusion along posterolateral sites of previously implanted MAGEC rods.

**Figure 7:**
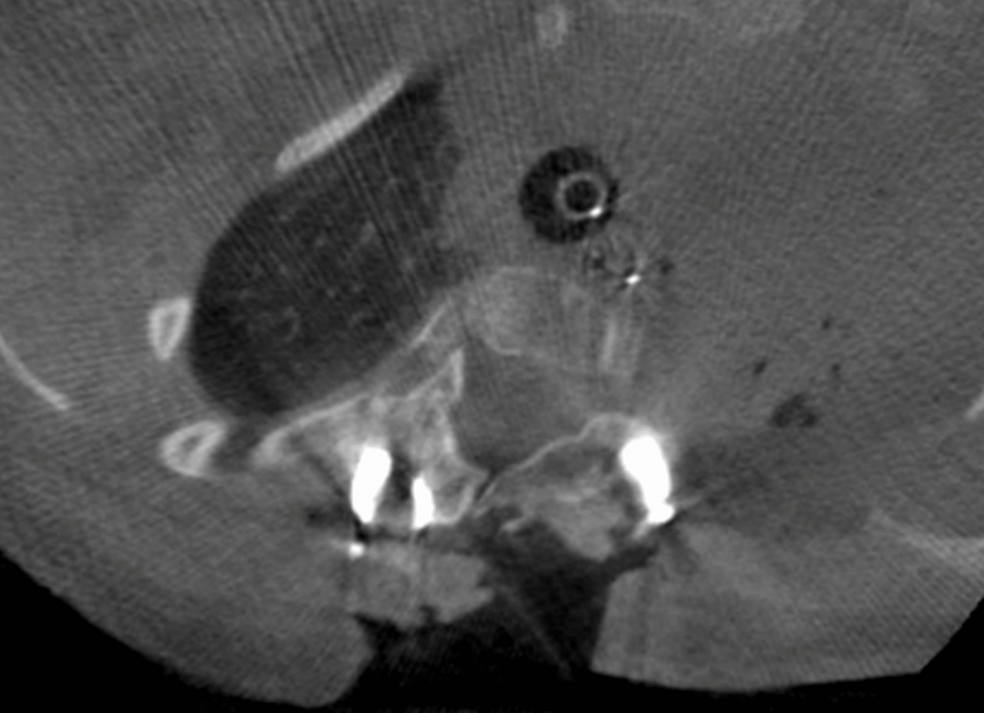
Inferior axial intra-operative CT image illustrating heterotopic bony fusion along posterolateral sites of previously implanted MAGEC rods.

Autofusion in patients with MCGRs is a rare occurrence, with only two reported cases in the literature. The first case involved a patient with Ehlers-Danlos Syndrome who underwent multiple reoperations, while the second case was observed in a Prader-Willi patient [18,19]. In this report, we present a unique case of autofusion in a patient with tetraplegic cerebral palsy and right thoracic neuromuscular scoliosis, who had undergone placement of dual MCGR instrumentation. Figures 6, 7 illustrate the heterotopic bone formation along the rostral aspect of the paths of the bilateral MCGRs. It is unclear what patient or surgical factors may induce autofusion; there were no instances of stalled lengthenings as confirmed radiographically. We postulate that autofusion is less common with MCGRs compared to TGRs for two primary reasons: (1) fewer surgeries result in less trauma to posterior spinal elements and musculature, and (2) more frequent MCGR lengthenings of smaller magnitude maintain a more constant, long-term distractive force [20]. We ultimately advocate for surgeon vigilance, such that autofusion is not ruled out in patients with MCGRs who demonstrate stalled lengthening or growth stagnation with progressive scoliotic deformity.

## Conclusions

The benefits of MCGRs make them an appealing alternative to TGRs for the treatment of EOS. Although MCGRs theoretically have a lower rate of autofusion compared to TGRs, we highlight a unique case of autofusion at the time of MCGR explantation and posterior spinal fusion and thus recommend further research into the use of MCGRs. Autofusion should not be overlooked as a possible complication of MCGRs used in the treatment of progressive EOS.

## Figures and Tables

**Figure 8:**
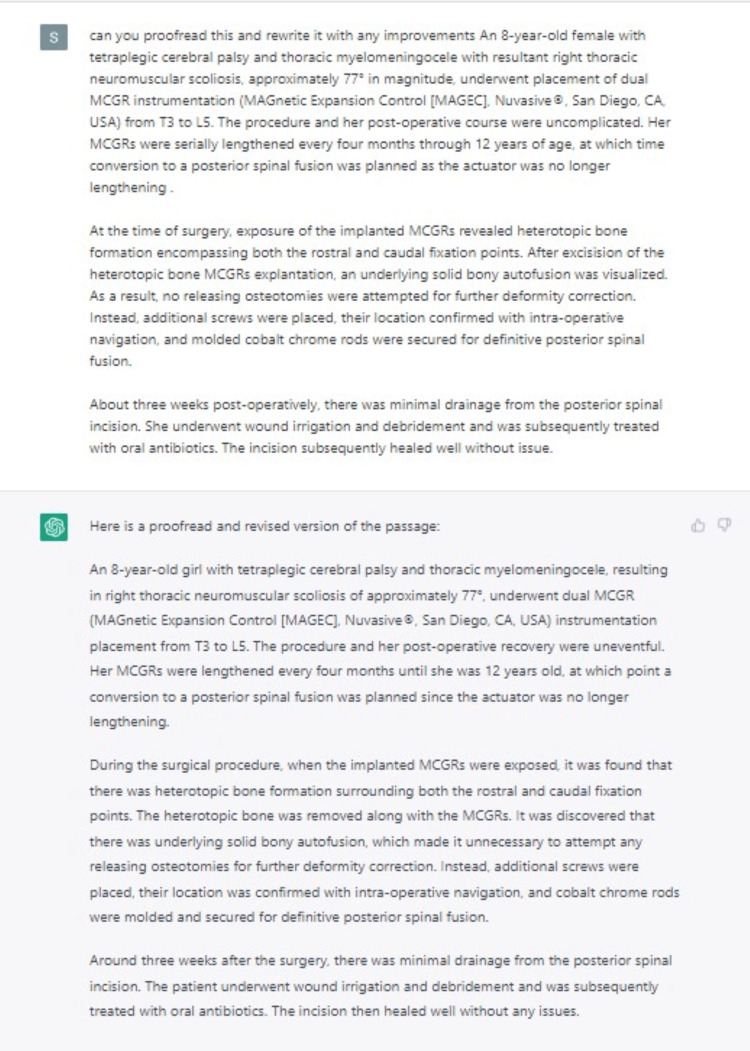
ChatGPT Initial Prompt

**Figure 9:**
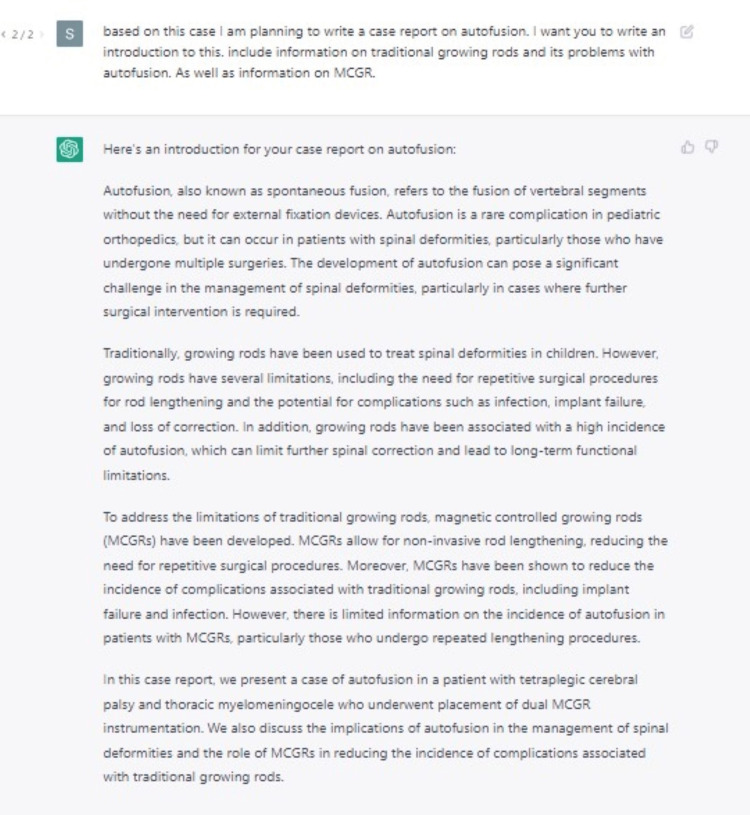
ChatGPT Second Prompt
